# Educational Case: Compare and contrast osteomalacia and rickets with respect to pathogenesis and clinicopathologic features

**DOI:** 10.1016/j.acpath.2024.100144

**Published:** 2024-09-05

**Authors:** Sarah Bunker, Judy S. Blebea, Jyotsna Pandey

**Affiliations:** Central Michigan University College of Medicine, Mount Pleasant, MI, USA

**Keywords:** Calcium metabolism, Musculoskeletal system, Non-neoplastic disorders, Organ system pathology, Osteomalacia, Pathology competencies, Rickets, Vitamin D deficiency

The following fictional case is intended as a learning tool within the Pathology Competencies for Medical Education (PCME), a set of national standards for teaching pathology. These are divided into three basic competencies: Disease Mechanisms and Processes, Organ System Pathology, and Diagnostic Medicine and Therapeutic Pathology. For additional information, and a full list of learning objectives for all three competencies, see https://www.journals.elsevier.com/academic-pathology/news/pathology-competencies-for-medical-education-pcme.^1^

## Primary objective

Objective MS2.1: Osteomalacia and Rickets. Compare and contrast osteomalacia and rickets with respect to pathogenesis and clinicopathologic features.

Competency 2: Organ System Pathology. Topic: Musculoskeletal System (MS). Learning Goal 2: Non-neoplastic Disorders of the Musculoskeletal System.

## Patient presentation, 1

A 6-month-old child presents for a routine well-child exam after being adopted by a family in Northern Minnesota. He arrived in the United States two weeks ago after living in Saudi Arabia with his birth mother and four older siblings for his first few months of life. On inquiry, the adoptive family says that they do not have any information about the health of his birth family. He was given up for adoption and stayed with the adoption-care agency until arriving in the USA. His adoptive parents are concerned because he has not had a bowel movement for the past two days and, compared to their other children, he seems to be small for his age. They report that he is unable to roll from his stomach to his back and he does not push up on his elbows when laying down. At times, he seems to have difficulty holding his head steady. They have occasionally noticed jitteriness and twitching in his legs and arms, especially when he cries, which are starting to concern them. He drinks 6–8 ounces of formula 4–5 times per day. He typically passes stool once every two days. His mother states he tends to be very fussy prior to having a bowel movement.

## Diagnostic findings, Patient 1, Part 1

On physical examination the infant is interactive with his environment, often reaching for objects. He coos and smiles energetically when spoken to. His weight is 6407 g, and his length is 65 cm. This puts him below the 5th percentile for weight and 25th percentile for height for age. He is unable to keep his head up when in a prone position and, when held, struggles to push down on his legs on a sturdy surface. Other physical exam findings include bilateral enlargement of the wrists and a slight pigeon chest. His anterior fontanel is enlarged and when palpated has a crackling feel. There appears to be a bossing of the frontal bones. The abdomen appears distended but is soft on palpation and there is no guarding or rebound tenderness.

## Questions/discussion points, Patient 1, Part 1

### What is the differential diagnosis?

The child is small for age and presents with symptoms suggestive of seizure or tetany, such as rhythmic twitching. Seizures could be due to brain infection (meningitis or encephalitis), cerebral tumors, or bleeding. Based on history and examination these conditions are less likely. Metabolic disturbances such as hypoglycemia, and electrolyte disorders such as hyponatremia or hypocalcemia should also be considered. Breastfed infants without supplementation can have a deficiency of vitamins A, D, K, and C.^2^, ^3^, ^4^, ^5^ However at this point, we are not seeing overt classic features of deficiency of vitamin A (dry skin, dry eye, Bitot spots, night blindness), vitamin K (a disorder of hemostasis- bleeding diathesis), and vitamin C (scurvy). Deficiency of vitamin D can lead to his clinical picture and is a possibility especially if there is attendant hypocalcemia. Hypocalcemia is a concern in infants that present with seizures, jitteriness, and periodic twitching. Low calcium levels can lead to carpal and pedal spasms, muscle cramping, and seizures.^6^ It is suggested that any infant that presents with hypocalcemic seizures should be screened for rickets as well as obtain an ECG and chest radiographs to evaluate for cardiac abnormalities and to rule out genetic causes such as DiGeorge syndrome.^7^ Clinical features of hypomagnesemia are similar to those of hypocalcemia. Another electrolyte to keep in mind is phosphate. Hypophosphatemia can present with seizures and chronic constipation.^8^ Low phosphate levels present with muscle weakness and pain, which could explain the infant's inability to hold his head up when in the prone position or support himself when his legs are on a stable surface.^8^

This patient's motor developmental delay and skeletal abnormalities suggest a musculoskeletal pathology. He has widened fontanelles with thinning of the bone (suggested by the crackling on palpation), and wrist enlargement with pigeon chest could be early indicators of rickets. The frontal bossing seen may be an additional feature of rickets especially in the light of the clinical features suggestive of hypocalcemia. Hungry bone disease is a rare disorder, defined as profound and prolonged hypocalcemia, hypophosphatemia, and hypomagnesemia that present with similar features. Hungry bone disease could be precipitated if the child was solely breastfed before adoption, as adding the phosphate and vitamin D-rich formula to his diet would result in rapid mobilization of calcium and phosphate to bone leading to hypocalcemia and the clinical picture seen.

### Based on the physical examination findings, what would be the next step(s)?

The child should be investigated for nutritional deficiencies and disorders that can lead to rickets and complications such as hyperparathyroidism. Laboratory tests for serum glucose, electrolytes, serum calcium, phosphate, magnesium, and alkaline phosphatase should be done. Renal function tests should also be included to assess renal health. Parathyroid hormone (PTH) should be included to assess for hyperparathyroidism.

Vitamin D levels should be determined as rickets is suspected. Only the 25-hydroxy form of vitamin D is routinely measured in suspected vitamin D deficiency. In the suspected genetic form of vitamin D-dependent rickets, 25-hydroxyvitamin D may be normal; however 1-25-hydroxyvitamin D would be low. This test should be included in the workup of this patient due to the age of the child and the absence of birth family history.

Obtaining bone radiographs is the appropriate next step to better understand the pathology by visualizing the growth plates. The patient's bilateral enlargement of the wrists, pigeon chest, frontal bossing, and crackling cranial bones are highly suggestive of rickets. Knee and wrist radiographs of a patient with rickets will show splaying, fraying, cupping, and course trabecular patterns of the metaphysis as well as a widening of the growth plate.^9^ Cortical spurs at right angles to the metaphysis and periosteal reaction may also be seen.^10^ Pseudofractures also known as Looser zones are radiolucent transverse bands that have a sclerotic border and are associated with rickets and osteomalacia.^9^ The radiolucent line of a pseudofracture is perpendicular to the long axis of the bone^11^ and is caused by deficient mineralization of the bone.^12^ These Looser zones are most often observed in the subtrochanteric region of the femur or metatarsals^12^ as the infant starts crawling and standing. Symmetric biconcavity of the vertebrae, called cod fish vertebrae, may also be seen in patients with rickets. Although these radiological findings may point toward rickets, a bone biopsy is the diagnostic gold standard for this pathology.^13^ A bone biopsy would show the specific pathologic abnormalities in bones that lead to the described radiologic features. Bone pathology is discussed in a later section. However, it is emphasized here that bone biopsies are rarely performed to confirm the diagnosis. The diagnosis is routinely made based on clinical features and radiologic findings.

## Diagnostic findings, Patient 1, Part 2

**Fig. 1:**
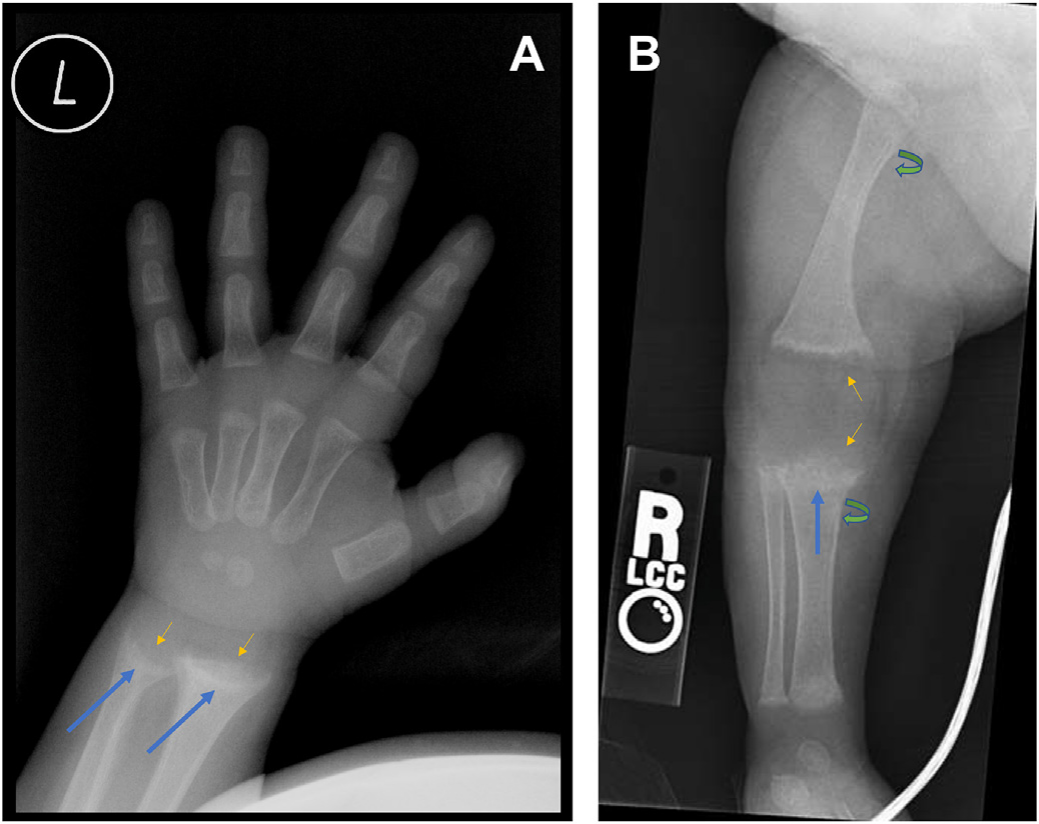
The features are consistent with rickets. In the growing skeleton, deficiency of normal mineralization of the provisional zone of calcification results in fraying and indistinct margins of the metaphysis (short thin yellow arrow), splaying and widening of the metaphyseal ends with cupping or concavity of the metaphysis (long blue arrows) seen in X-rays of both the hand (A) and the knee joint (B). These features are most prominent in the wrist and knee and are also seen at anterior rib ends where they are described as rachitic rosary (not shown). Diffuse periosteal reaction may also be seen along the distal radius and ulna, distal femur, and proximal tibia (curved arrows). Mild bowing of the femur and tibia can occur and is seen in the radiographs (Fig. A: Case courtesy of Dr. Hani Makky Al Salam, Radiopaedia.org, rID: 8971 https://radiopaedia.org/cases/8971 and Fig. B: Case courtesy of The Radswiki, Radiopaedia.org, rID: 11889 https://radiopaedia.org/cases/11889).

The laboratory investigations are detailed in Table 1. The X-rays for wrists and knee joints are done and the findings of widening and cupping of the metaphysis of long bones are identified, as shown in are depicted in Fig. 1A and B.

**Table 1:** Laboratory investigations for the child.

Test	Patient value	Reference range
Glucose (mg/dL)	106	70–110
Urea Nitrogen (mg/dL)	6	5–20
Creatinine (mg/dL)	0.3	0.3–1.0
Total protein (g/dL)	6.2	6.0–8.3
Albumin (g/dL)	3.7	3.3–5.0
Globulin (g/dL)	2.3	2.6–4.1
Sodium (mmol/L)	135	135–145
Potassium (mmol/L)	3.4	3.4–4.8
Chloride (mmol/L)	104	98–106
Magnesium (mg/dL)	2.1	1.7–2.4
Calcium (mg/dL)	6.2	8.5–10.5
Ionized Calcium (mmol/L)	0.85	1.14–1.30
Phosphorus (mg/dL)	3.0	3.3–5.0
Alkaline Phosphatase (U/L)	854	15–350
25-Hydroxyvitamin D (ng/mL)	6	20–80
1,25-Hydroxyvitamin D (pg/mL)	15	24–86
Parathyroid hormone (PTH) (pg/mL)	125	10–60

## Questions/discussion points, Patient 1, Part 2

### Interpret the patient's laboratory results. What is the diagnosis?

There is marked hypocalcemia and both total and ionized calcium levels are low. Low 25-hydroxyvitamin D levels are found and suggest hypovitaminosis D. PTH is increased along with high alkaline phosphatase. The laboratory results suggest secondary hyperparathyroidism and attendant increased bone remodeling. These findings along with the X-ray findings confirm a final diagnosis of rickets due to vitamin D deficiency.

### Describe the histologic features of bone in this disorder as shown in Fig. 2, Fig. 3, Fig. 4 taken from a different patient. How would these findings change with treatment (Fig. 5)?

**Fig. 2:**
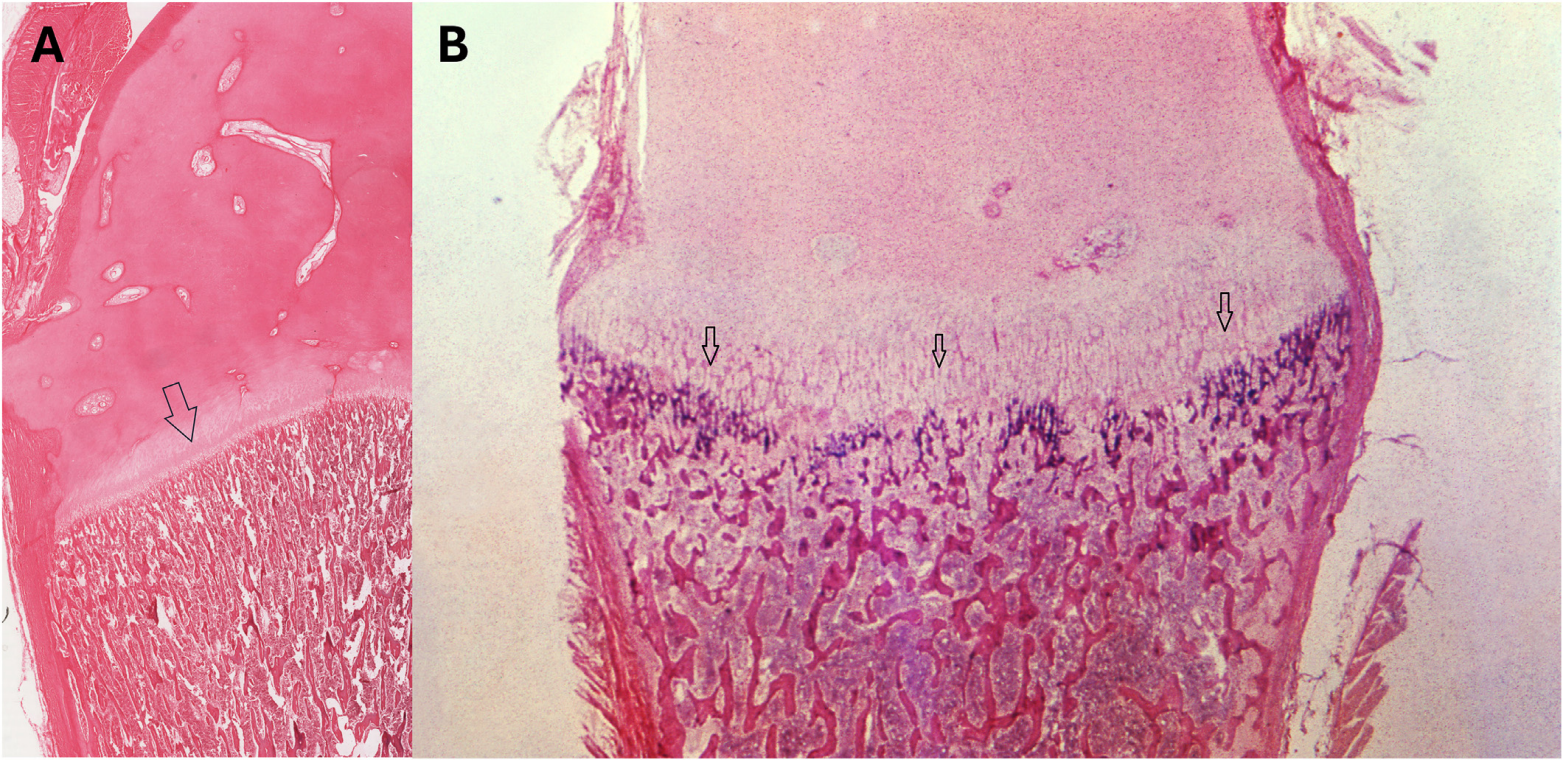
(A) The photomicrograph (low power) the arrow points to the normal growth plate (Image courtesy: Regents of the University of Michigan, licensed under Creative Commons Attribution-Noncommercial-Share Alike 3.0 License). (B) The photomicrograph (low power) shows the growth plate of a patient with rickets. The salient histopathologic changes seen suggest that the zone between cartilage and bone is cytoarchitecturally disorganized, with a haphazard arrangement of cells. Invasion of numerous blood vessels can also be seen (arrow) (Image Courtesy: Image #21272, Public Health Image Library, CDC. https://phil.cdc.gov/Details.aspx?pid=21272).

**Fig. 3:**
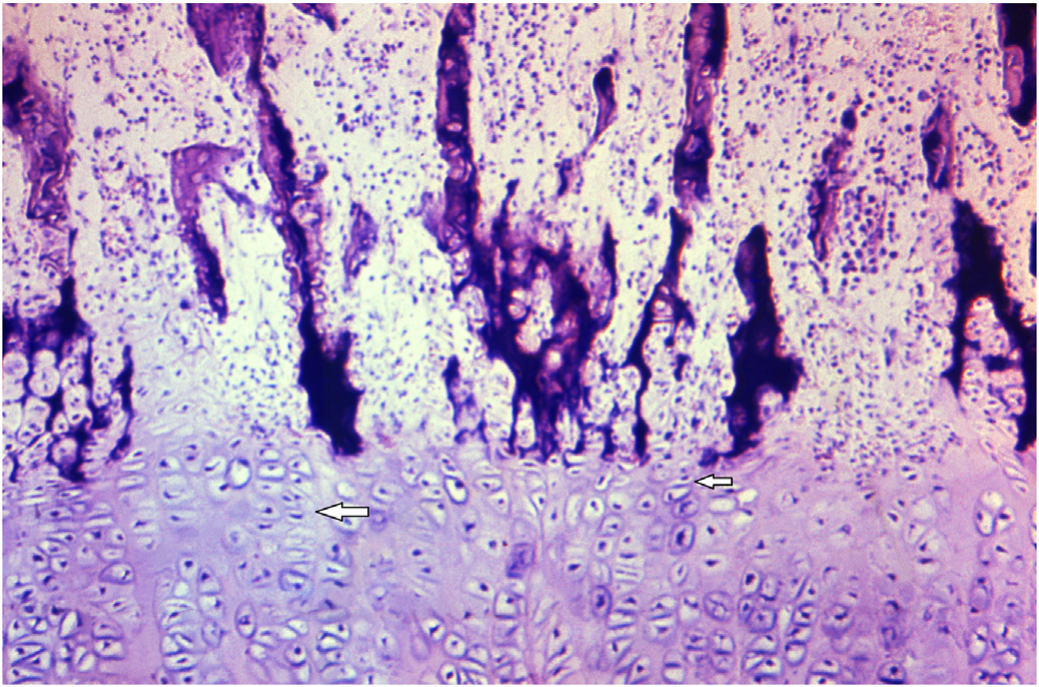
The photomicrograph (high power) shows an early lesion of rickets. The section is taken from the growth plate through the region of the epiphyseal plate. There is decreased deposition of inorganic minerals in the cartilaginous tissue, between the hypertrophic columns of chondrocytes (arrow). Additionally, there is beginning of disorganization of the columnar growth pattern of the chondrocytes (Image Courtesy: Image #21271, Public Health Image Library, CDC. https://phil.cdc.gov/Details.aspx?pid=21271).

**Fig. 4:**
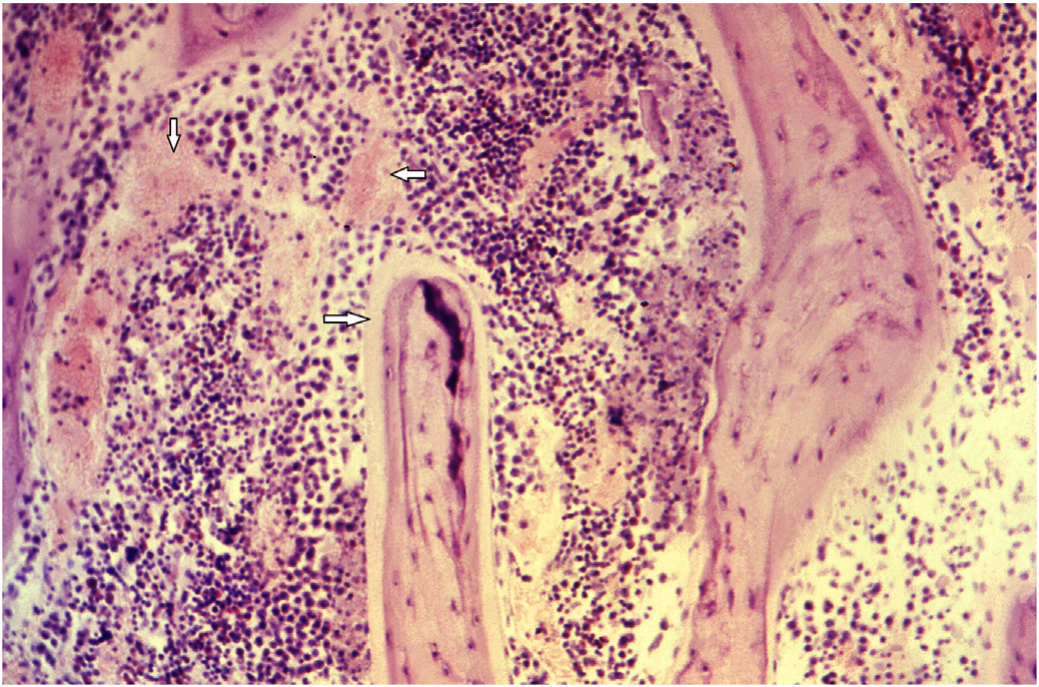
The photomicrograph (medium power) shows prominent areas of osteoid formation in a patient with rickets and the trabeculae within the bone shaft are poorly calcified due to the poor mineralization process (white arrows) (Image Courtesy: Image #21273, Public Health Image Library, CDC. https://phil.cdc.gov/Details.aspx?pid=21273).

**Fig. 5:**
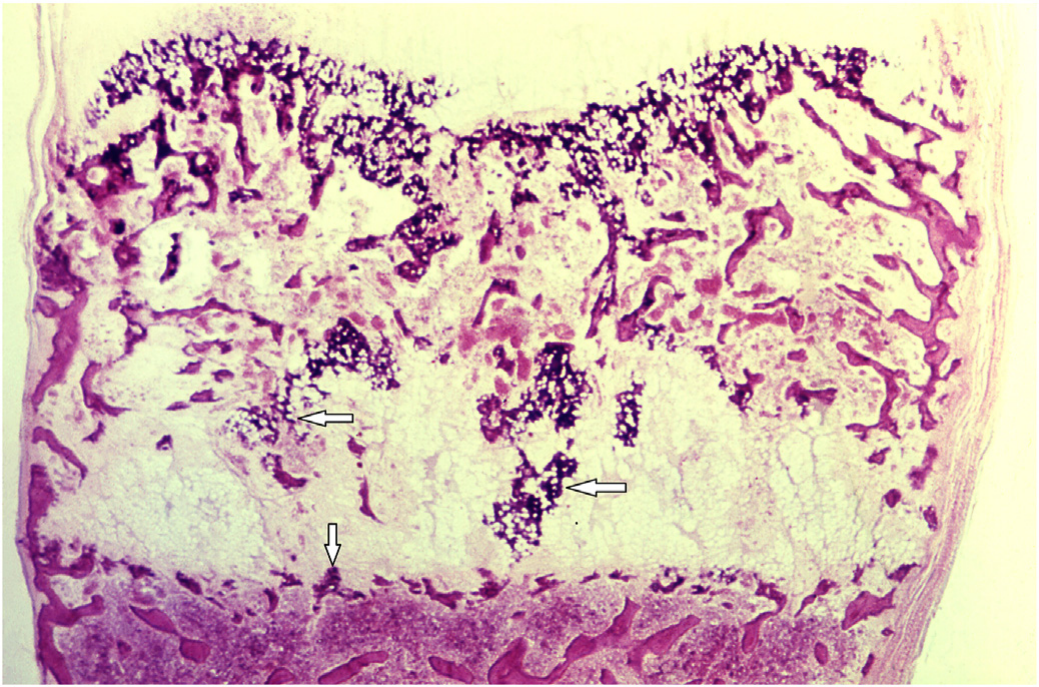
The photomicrograph (low power) shows post-therapy changes in the bone of a patient of rickets treated with vitamin D. The bone's mineralization process seen as zones of enchondral ossification has restarted within the cartilaginous matrix. There are areas of irregularly shaped, more darkly stained developing trabeculae (arrow), that can be seen between the distal epiphysis and proximal metaphysis (Image Courtesy: Image #21275, Public Health Image Library, CDC. https://phil.cdc.gov/Details.aspx?pid=21275).

Bones in a child with rickets have defective mineralization of the bone and the cartilaginous matrix. The growth plate would be thickened and reveal disorganization along with a large zone of hypertrophic cartilage, although difficult to appreciate at low magnification on histopathology (Fig. 2). As the woven bone laid down on the surface of the cartilage is poorly mineralized there is failure to remodel the metaphysis. Thus, in rickets, the failure to mineralize the matrix of epiphyseal cartilage additionally prevents normal osteoclastic resorption of the cartilage. This leads to a thickened and poorly defined hypertrophic zone (Fig. 3). Osteoclastic giant cells may be seen penetrating the poorly mineralized cartilage plate in a haphazard or irregular pattern. The adjacent primary trabeculae may be unmineralized or poorly mineralized. Some of these changes are seen in Fig. 4. There may be hemorrhage at this interface of the growth plate and metaphysis. This hemorrhage is a result of microfractures of the poorly mineralized trabeculae. There is a reversal of these features with treatment leading to enchondral ossification and development of trabeculae within the physis proximal to the metaphysis (Fig. 5).

### Describe normal calcium, phosphate, and vitamin D metabolism and their regulators

Calcium, phosphate, and vitamin D all play vital roles in bone health. Vitamin D_3_ amounts to 90% of the body's vitamin D stores and is formed naturally in the skin when exposed to sunlight, whereas vitamin D_2_ is mainly absorbed through the gut from dietary sources, such as milk and dairy products, fish, eggs, plants, and grains.^14^ Both vitamin D_3_ and D_2_, collectively known as vitamin D, enter the blood. In the liver, the 25-hydroxylase enzyme converts vitamin D to 25-hydroxyvitamin D. 25-hydroxyvitamin D is transported to the kidney and is converted to 1,25-dihydroxyvitamin D by the 1-α-hydroxylase enzyme. Both 25-hydroxyvitamin D and 1,25-hydroxyvitamin D are biologically active forms of vitamin D. However, 1,25-hydroxyvitamin D is the more potent of the two.^14^ It plays many roles throughout the body, such as increasing calcium absorption in the intestine and the distal tubules of the kidney and bone calcification.^14^ The conversion of 25-hydroxyvitamin D to 1,25-hydroxyvitamin D is regulated by several mechanisms that influence the production of 1-α-hydroxylase. The parathyroid gland is highly sensitive to blood calcium levels. Falling blood calcium concentration triggers the release of PTH which upregulates 1-α-hydroxylase production in renal tubules. This results in the upregulation of the conversion of 25-hydroxyvitamin D to 1,25-hydroxyvitamin D resulting in increased absorption of calcium from the gut and also resorption of calcium in the renal distal tubules. This effectively increases blood calcium levels. Mechanistically, this increase in blood calcium is not just by increased absorption in the intestines but also through regulated bone remodeling activity. 1,25-hydroxyvitamin D acts both directly and indirectly on the bones. It stimulates osteoblastic activity that includes stimulation of RANK-L and activation of RANK receptors on osteoclast precursors.^14^ Activation by RANK allows for osteoclast maturation, ultimately releasing calcium and phosphate into the bloodstream to be used for bone mineralization and metabolic functions throughout the body.^14^ 1,25-hydroxyvitamin D downregulates itself via a negative feedback mechanism by shutting off renal 1-α-hydroxylase enzyme. 1,25-hydroxyvitamin D also increases phosphate absorption in the gut, and the increased bone remodeling releases phosphate along with calcium into the blood. As a normal physiologic response, an increased level of phosphate triggers PTH release, causing increased phosphate excretion by the kidney. Increased levels of phosphate also trigger FGF23 release from osteocytes. FGF23 has a negative regulatory effect on both the parathyroid gland and renal 1-α-hydroxylase enzyme. It directly acts to increase phosphate excretion in the tubules creating a regulatory loop for itself. Additionally, adequate levels of vitamin D have a mild inhibitory effect on parathyroid gland and PTH production. Thus, there is a tight regulation of each electrolyte (calcium and phosphate), hormone (PTH, FGF23), and vitamin D's effects. Functionally 1,25-hydroxyvitamin D, or calcitriol, can be thought to bring about the retention of sufficient mineral ions to ensure mineralization of bone matrix, whereas PTH maintains the proper ratio of calcium to phosphate in plasma and interstitial fluid so that normal physiologic function is maintained.

### How does vitamin D deficiency lead to the clinical features associated with rickets?

In vitamin D-deficient states, there is deficient calcium absorption in the gut and inadequate retention of calcium by the kidney. This produces a hypocalcemic state. Similar hypocalcemia would be produced if there was a dietary deficiency of calcium. When calcium levels are low, there is an increase in PTH production that then activates 1-α-hydroxylase in the renal tubules to increase the amount of active vitamin D. However, this is not possible when hypovitaminosis D causes sustained hypocalcemia leading to a more sustained increase of PTH. PTH now mobilizes calcium from the bone and decreases renal calcium excretion in an attempt to normalize blood calcium levels.^14^ As the calcium is mobilized from the bone it leads to increased phosphate in blood. Hyperphosphatemia causes FGF23 release from osteocytes leading to a decrease in phosphate resorption and active excretion of phosphate by the kidney causing hypophosphatemia. FGF23 also suppresses 1-α-hydroxylase, intensifying the unavailability of biologically active vitamin D. So, the net effect of hypovitaminosis D or dietary deficiency of calcium is hypocalcemia, hypophosphatemia, decreased osteoblastic activity, and increased levels of PTH. There is also increased osteoclastic activity due to the increase in PTH levels which leads to a higher turnover rate in adults and poor calcification in children. Thus, in patients with rickets, lack of adequate vitamin D or calcium causes inadequate calcification of cartilage cells, leading to an overgrowth of epiphyseal cartilage and persistence of distorted cartilage that projects into the bone marrow cavity, leading to a poorly organized osteoid matrix, ultimately leading to the characteristic enlargement of joint spaces and loss of structural integrity of the bone^14^ producing the clinical features associated with rickets.^15^

### As this patient gets older, what other clinical features can be expected if treatment is not initiated?

As infants age and grow, their growth plates are constantly remodeling and, as a result, more skeletal abnormalities will be observed. One of the early signs of subclinical vitamin D deficiency, especially in newborns born to vitamin D deficient mothers and/or solely breastfed, is craniotabes, a softening of the outer table of the skull bones. However, this finding can be seen in several other disorders such as osteogenesis imperfecta, hydrocephalus, congenital syphilis, and hypervitaminosis D, just to name a few important pathologies. If craniotabes is seen in an otherwise normal infant, the possibility of vitamin D deficiency and early rickets should be explored.^16^^,^^17^ Infants with rickets present with many cranial abnormalities such as flattened occipital bones, buckled parietal bones, a squared facial appearance, and frontal bossing.^14^ They will also have an outward protrusion of the sternum, also described as pigeon chest. Depression of the sixth and seventh costal cartilage known as Harrison groove may also be seen.^13^ By one year of age, the rachitic rosary may be seen due to expansion of the costochondral junctions by overgrowth and persistence of cartilage. By two years of age kyphoscoliosis and a narrow pelvis can be expected.^13^ Prominent long bone deformities, such as genu varum, and prominent enlargement of the wrists and costochondral junctions manifest during rapid growth phases as the bones are rapidly laying down cartilage and attempting to mineralize.^18^ Typically, genu varum manifests when infants start to walk. It is defined as a femoral intercondylar distance of more than five centimeters.^12^ As children mature and move past rapid growth phases, they may present with motor delay, proximal myopathy, and dental complications.^9^ Decreased calcium levels, as seen in patients with rickets, lead to altered cellular energy, resulting in a decreased ability for the muscle fibers to fire efficiently, leading to myopathy and poor muscle tone. Recurrent dental abscesses and abnormal tooth morphology in patients with rickets are due to vitamin D deficiency and, more importantly, abnormal thin globular dentin.^19^ This leads to enlarged pulp horns in the gums that extend into the dentin-enamel junction and allow for invasion of the pulp by microorganisms and toxins with an attenuated immune response to said infection.^19^ Dental changes are so frequent in patients with rickets that some patients first get the diagnosis of rickets after a visit to the dentist due to recurrent abscesses.

### Discuss the treatment recommendation for a patient with vitamin D-deficient rickets

Treatment of rickets includes supplementation with vitamin D and increased sun exposure.^11^ Vitamin D does come in both D_2_ and D_3_ forms, the most commonly used supplementation in the United States being calcitriol or 1,25-hydroxyvitamin D.^11^ Patients with rickets should get both vitamin D and phosphate replacement, whereas patients with hypophosphatemia or hereditary rickets that lack renal 1-α-hydroxylase activity should be treated with calcitriol and oral phosphate.^13^ Resolution of symptoms may occur in a few weeks to months. However, radiological and histological findings will take several months to years to resolve. Therefore, supplementation should not be stopped as soon as the patient is asymptomatic. Every 1–3 months, patients with rickets should have blood work done to evaluate their calcium, phosphate, alkaline phosphatase, and PTH values.

## Patient presentation, 2

One year later, the child's birth mother aged 28 years, who still resides in Saudi Arabia, reached out to the adoption agency because she was pregnant again and asked if the same family would like to adopt another child. The adoptive parents are interested and because of the child's rickets diagnosis, his adopted parents decide that it is important to have the mother screened for vitamin D and calcium deficiencies. A consultation with a clinic in her village is scheduled. This is the birth mother's sixth child. She has breastfed all her children. She spends most of her days doing housework inside and caring for her children. When she does leave the house, she is heavily veiled due to religious practices. The family's diet includes primarily chicken, potatoes, and rice. On inquiry, she admits to having muscle aches and bone pain. She often feels getting up from a seated position or reaching overhead is increasingly difficult. She also complains of increased fatigue but thinks it is pregnancy-related.

## Diagnostic findings, Patient 2, Part 1

On a focused examination of the musculoskeletal system, the mother has notable genu varum and mild kyphoscoliosis. Her bone pain is localized to the lower back and hips. The physical exam is otherwise unremarkable. She is 12 weeks pregnant with the corresponding expected uterine size.

The physician decides on further laboratory investigations based on the patient's physical exam and personal history. X-rays are not ordered as she is pregnant. The laboratory investigations are detailed in Table 2.

**Table 2:** Mother's laboratory investigations.

Parameters	Patient value	Reference range
Calcium (mg/dL)	8.0	8.5–10.5
Phosphate (mg/dL)	2.5	3.3–5.0
25-Hydroxyvitamin D (ng/mL)	8	20–80
PTH (pg/mL)	140	10–60
Alkaline phosphatase (U/L)	422	15–350
Creatinine (mg/dL)	0.8	0.6–1.3
Albumin (g/dL)	4	3.3–5.0
Estimated GFR (mL/min/1.72 m^2^)	80	60–120

## Questions/discussion points, Patient 2, Part 1

### What is the mother's diagnosis?

Based on the patient's physical exam and personal history, the suspicion of osteomalacia is strong. The laboratory investigations support a diagnosis of vitamin D deficiency. Based on the clinical findings, the diagnosis is osteomalacia due to vitamin D deficiency.

### What is the difference between osteomalacia and rickets? How are they different from osteoporosis and osteopenia?

Osteomalacia is seen in adults whereas rickets is a disease of growing children when vitamin D deficiency is present. Vitamin D deficiency is the most common cause of osteomalacia in adults. Both osteomalacia and rickets are characterized by inadequate mineralization of bone. Osteomalacia or “soft bones” is seen in adults who have vitamin D metabolism disorders and is characterized by inadequate mineralization of bone matrix and accumulation of osteoid.^10^ Rickets affects bone development in children in whom growth plates (physes) are open. It is characterized by defective mineralization of bone as well as defects of the cartilaginous matrix of the growth plate. Both these disorders cause bone pain, waddling gait, and soft, weak bones that can lead to bone deformities and poor growth (children).^11^ Osteoporosis on the other hand is a distinct condition defined as decreased bone mineral density and decreased bone mass. There may be changes to the structure of the bone due to excessive demineralization. There is decreased bone strength that increases the risk of pathological fractures.^20^ Although it can occur at any age, the risk increases with age and may occur especially in post-menopausal women. Several medications may also increase the risk of osteoporosis e.g., glucocorticoid steroids.^20^ Early onset osteoporosis is part of the differential diagnosis for osteomalacia, and it is important to differentiate between the two. A clear and accurate distinction between the two can be made by bone biopsy with double tetracycline labeling.^21^ In practice, however, tetracycline labeling is not used. The diagnosis is instead confirmed by combining the cumulative findings on the history, physical examination, radiological evaluation, and laboratory results. Patients with osteoporosis will have normal levels of serum calcium, phosphate, alkaline phosphatase, and PTH, whereas abnormalities in at least one of these laboratory values are commonly seen in osteomalacia. Osteopenia also refers to decreased bone mineral density, but bone mass loss has not yet developed. It may be seen as an in-between condition from normal mineralized bone to fully developed osteoporosis.^22^

### What is the typical history, physical examination findings, and laboratory investigations that confirm osteomalacia?

The clinical features of osteomalacia are general and non-specific with many patients remaining asymptomatic for years. Patients present with bone pain, muscle weakness, and occasionally with fragility fractures. Bone pain is often symmetrical and bilateral, most often aggravated by weight bearing or muscle contractions while walking.^11^^,^^23^ Pain typically begins in the lower back and spreads to the pelvis, hips, thighs, and upper back, which explains why our patient could have blamed her pregnancy on the pains and fatigue she was experiencing. Bone pain is often dull, diffuse, and poorly localized; it may be misdiagnosed as fibromyalgia due to the widespread and non-specific distribution. This pain is rarely felt below the knees unless there is a fracture or pseudo-fracture present.^11^^,^^23^ Pain can be elicited with pressure or percussion over the bone and may be relieved with stretching. Walking may be difficult for patients with osteomalacia due to proximal muscle weakness, resulting in a waddling gait.^11^^,^^23^ Skeletal deformities are rare in adults; they are more often seen in children with rickets.^11^ When skeletal deformities do occur in adults, they are often due to the presence of pre-existing fractures from the weakened nature of the bone.^11^^,^^23^ Considering the patient's laboratory investigations that reveal low vitamin D levels, geographic location, and religious practices, along with her extensive time indoors and multiparity, it is likely that she is suffering from osteomalacia. The son that she put up for adoption was diagnosed with rickets. It is well-recognized that the fetal bone development is dependent on maternal calcium and vitamin D levels. Thus, when mothers have low vitamin D stores, it is likely that the child would be predisposed to rickets. Because rickets and osteomalacia are so closely related in both pathogenesis and risk factors, the main lab investigation findings are low vitamin D levels and variable calcium and phosphate levels^13^ in both conditions.

### What would be the expected findings if the radiological investigation had been done?

**Fig. 6:**
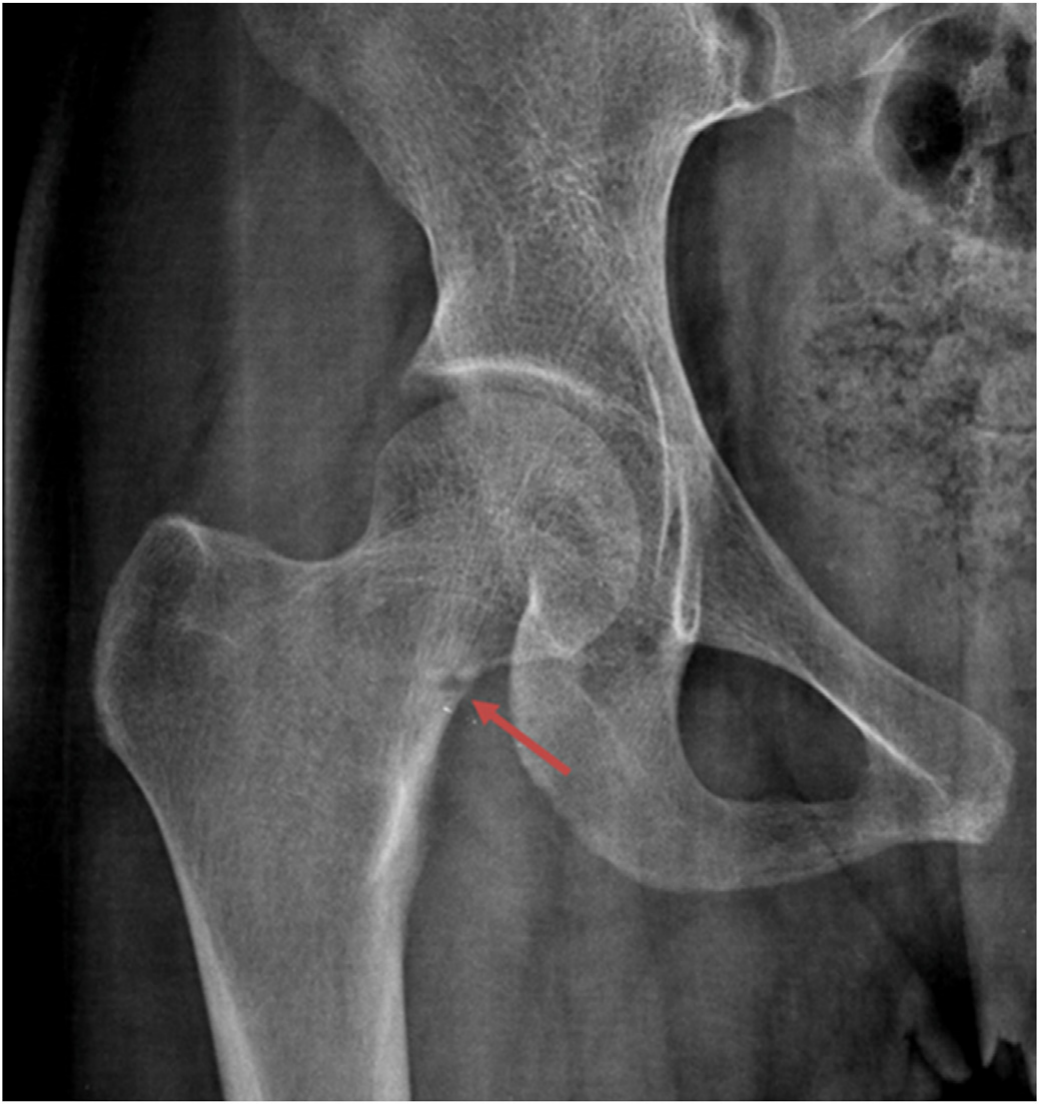
The radiograph depicts Looser zone involving the right medial femoral neck in a patient with osteomalacia (red arrow). Case courtesy of Dr. Maulik S Patel, Radiopaedia.org, rID: 16273. https://radiopaedia.org/cases/16273.

Unlike rickets, osteomalacia has mostly non-specific radiological features.^11^^,^^24^ Osteopenia may be seen on radiographic images similar to osteoporosis. While radiographs were not obtained in this pregnant patient, the classic radiographic finding of osteomalacia is the presence of radiolucent bands perpendicular to the cortex of the bone that results from focal deposition of uncalcified osteoid. These bands are referred to as Looser zones or pseudo-fractures. Osteomalacia should strongly be considered when the Looser zones are bilateral and symmetric and in a typical location such as the femoral neck, subtrochanteric region, superior and inferior pubic rami, axillary margins of the scapula, and ribs.^11^^,^^23^^,^^24^ An example of a Looser zone involving the right medial femoral neck in another patient with osteomalacia is given in Fig. 6.

### Discuss the pathogenesis of osteomalacia. Correlate the features with expected histopathologic findings

**Fig. 7:**
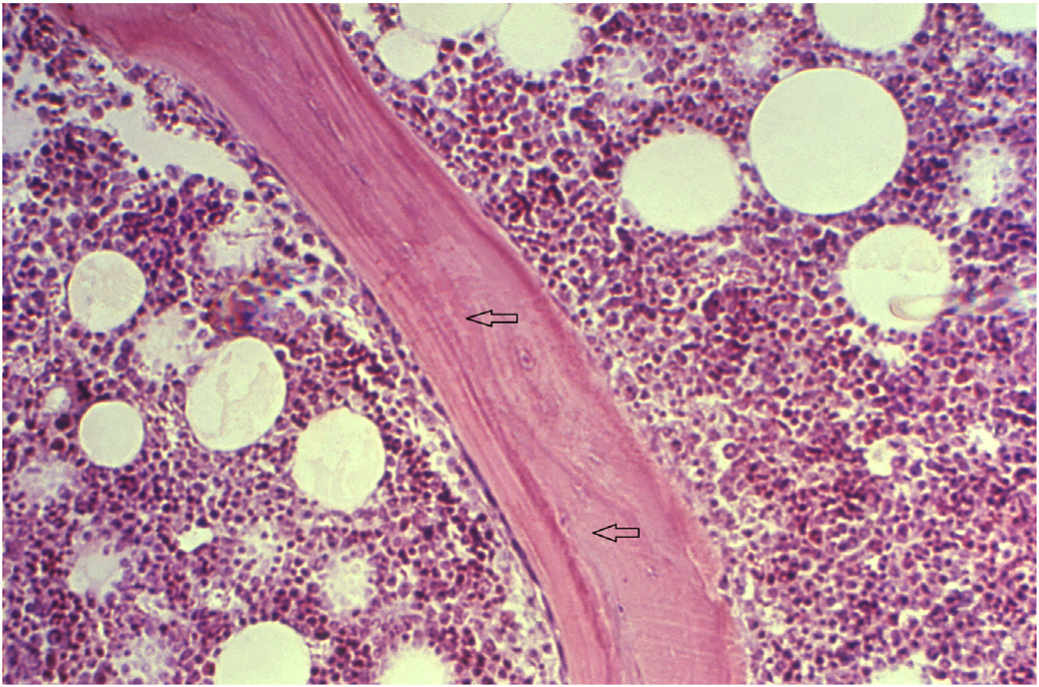
The photomicrograph (medium power) is taken from the bone of a patient with osteomalacia due to vitamin D deficiency. The main histopathological changes seen are deposits of osteoid that border the trabeculae (arrow) (Image Courtesy: Image #21276, Public Health Image Library, CDC. https://phil.cdc.gov/Details.aspx?pid=21276).

Like rickets, osteomalacia is due to a lack of vitamin D which causes a derangement of the normal bone remodeling process.^14^ However, an increase in cartilaginous matrix is a feature of rickets and is not seen in adults with osteomalacia. The pathology of osteomalacia on the other hand has in addition to deficient calcification, a delay in mineralization of bone matrix that leads to an increase in osteoid. Osteoid forms a thick seam that covers parts of the trabecular surface (Fig. 7). When there is a deficiency of vitamin D, osteoblasts are unable to adequately lay down and mineralize the osteoid matrix leading to a persistence of excess osteoid.^14^ Another consequence of low vitamin D levels is an excess secretion of PTH, leading to secondary hyperparathyroidism which further increases osteoid surface and volume, but not overall thickness causing a broadening.^11^ The unmineralized osteoid is weaker in comparison to well-formed bone and is more prone to fracture, especially in the vertebral bodies and femoral neck.^14^ At this stage, many patients are asymptomatic with normal calcium and phosphate levels.^11^ As osteoid surface and volume continue to increase due to hyperparathyroidism, thickness eventually increases as well with some preservation of bone mineralization.^11^ At this stage in the disease, patients often present with the classic clinical features of bone pain and muscle weakness.^11^ However, as the disease progresses mineralization of the bone matrix ceases while osteoid accumulation continues. Once osteoid accumulation reaches to cover more than 90% of the bone surface, the patients have progressed to the final stage of osteomalacia. Peri-trabecular and bone marrow fibrosis are two specific features of severe hyperparathyroidism that can eventually be seen in osteomalacia patients.^11^

### Enumerate the causal pathologies and disorders that may lead to osteomalacia and rickets

Both rickets and osteomalacia can be divided into two groups; due to hypovitaminosis D or due to disorders of phosphate metabolism that interfere with vitamin D metabolism. Resultant calcium and phosphate deficiencies can present in the same manner.^14^

Hypovitaminosis D could be acquired from (a) inadequate exposure to sunlight, (b) deficient dietary intake, and (c) defective intestinal absorption. Additionally, several other acquired or hereditary disorders can lead to abnormal vitamin D metabolism and hypovitaminosis D (Table 3). Similarly, impaired resorption of phosphates in the proximal renal tubules can lead to hypophosphatemia that results in impaired vitamin D metabolism and hypovitaminosis D. X-linked hypophosphatemia (XLH), a rare disorder, is the most common cause of inherited phosphate wasting due to a mutation in the *PHEX* gene that predominantly affects osteoblast activity and FGF23 production.^15^^,^^25^ Several pharmaceutical drugs, e.g., aluminum, fluoride, iron, and etidronate, can also affect mineralization and present a similar picture.

**Table 3:** Selected causes and disorders associated with rickets and osteomalacia.

Hypovitaminosis D		
Vitamin D deficiency	Dietary deficiency, Inadequate sun exposure	Both Children and adults- rickets, osteomalacia
Inadequate intestinal absorption	Malabsorption syndrome	Mainly seen in adults- osteomalacia
	Cholestatic liver disorders	
	Biliary obstruction	
	Chronic pancreatic insufficiency	
Inherited disorders of Vitamin D metabolism	1-α-hydroxylase deficiency	Vitamin D-dependent rickets Type I (Type I VDD)
	Target organ insensitivity to 1,25(OH)_2_D	Vitamin D-dependent rickets Type II (Type II VDD)
Acquired disorders of Vitamin D metabolism	Hypoparathyroidism	Osteomalacia
	Tumor-induced osteomalacia	
	Chronic renal failure (CRF)	
	Old age	
Disorders of phosphate metabolism		
X-linked hypophosphatemia	Dominant trait	Vitamin D resistant rickets
	*PHEX* gene mutation	
	Massive phosphate wasting	
Fanconi syndrome (Genetic)	Renal wastage of phosphate, glucose, bicarbonate, and amino acids	Renal tubular acidosis Osteomalacia or rickets
	Wilson disease	
	Tyrosinemia	
	Galactosemia	
	Glycogen storage disease	
	Cystinosis	
Fanconi syndrome (Acquired)	Paraneoplastic syndrome associated with benign or malignant soft tissue and bone tumors	Tumor-associated osteomalacia

### What lab investigations are used to differentiate different etiologies of rickets and osteomalacia?

Vitamin D level variation allows for differentiation of the various forms of rickets. Low serum levels of vitamin D are suggestive of acquired hypovitaminosis D or 1-α-hydroxylase deficiency (vitamin D dependent rickets type I) whereas normal serum levels of vitamin D suggest calcium deficiency or recent onset rickets. High serum vitamin D levels would be seen in vitamin D-dependent rickets type II,^11^ a rare autosomal recessive disorder. The main pathology in vitamin D-dependent rickets type II is end organ hypo-responsiveness to vitamin D. The clinical signs and symptoms manifest by one year of age. Although lab work and radiology are normally used for diagnostic indicators of rickets, the most reliable diagnostic test would be to obtain a bone biopsy as stated previously. However, bone biopsy is not routinely done in practice.

### What are the risk factors and prevention strategies for rickets and osteomalacia?

The risk factors of osteomalacia and rickets mirror each other, aside from the maternal aspect of passing vitamin D stores on to infants.^14^ The most at-risk population are dark-skinned ethnic minority groups especially those who live in Northern latitudes above 34° as the high melanin content reduces the dose of UV exposure and reduced daylight time periods of sun exposure overall.^9^ Once these populations have been identified, education is one of the key factors for prevention.^13^ All individuals should be aware that they should be getting daily sun exposure to increase vitamin D intake and should increase their dietary intake of dairy and fortified foods like eggs and milk.^9^ Some mushrooms and yeast are being fortified with UV enhancement to increase vitamin levels within these food groups.^13^ A combination of both supplementation and fortification is important for both the treatment and prevention of osteomalacia.^9^

The most important preventative measure is to screen at-risk populations. Patients at risk of rickets correlate with geography. Populations most at risk of rickets are those living in northern latitudes with less sunlight exposure, and those of religious practices that require women to wear heavy veils or avoid direct sun to limit skin darkening.^14^ In Saudi Arabia, 92% of girls, 79% of boys, and 75% of men and women are deficient in vitamin D.^9^ Likewise, in countries located above 34° latitude, there is an increased risk of rickets. 18% of the European population is vitamin D deficient and it has been deemed as a pandemic in the winter months.^9^ There is a 3–7 times higher risk of dark-skinned ethnic minority groups in the UK to be deficient in vitamin D and present with symptomatic rickets.^9^ Likewise, individuals on therapies such as antiretroviral therapy, anticonvulsant therapy, or antacids all have a higher risk of deficiencies, as do patients with renal tubular disorders and malabsorptive disorders.^11^ To prevent rickets, patients should start supplementation, be on a fortified diet, or both.^18^ There are also many fortification efforts, such as adding vitamin D into milk and dairy products, as well as to some livestock products such as eggs to increase daily intake.^18^ Any child above the age of 12 years, and any adult, should be taking a dose of 600 IU daily to ensure proper vitamin D stores, as well as calcium and phosphate supplementation to ensure proper metabolism and internal balance.^18^ Some sources suggest that ethnic groups should have supplementation initiated as soon as they arrive in the United States, and at-risk populations should be on a dose of 600 IU daily.^18^ Likewise, pregnant mothers should be on a daily dose of 600 IU to prevent deficiencies during pregnancy, because if they have low vitamin D stores, they do pass this deficiency on to their infant. For this reason, pregnant mothers should be screened and if the mother is deficient, newborns should have vitamin D supplementation initiated promptly.^18^ If a child has a history of calcium, phosphate, or vitamin D deficiency, all other children in that household should be screened for deficiencies as well.^18^ Individuals should also incorporate vitamin D-fortified foods, such as dairy and eggs, into their diets. A combination of both supplementation and fortification should be advised for at-risk populations to prevent both rickets and osteomalacia.

## Teaching points

• Both rickets and osteomalacia are disorders of impaired mineralization of the bone matrix. The most common cause for both rickets and osteomalacia is severe and prolonged vitamin D deficiency. Both conditions can also occur due to inherited defects of phosphate and vitamin D metabolism.
• The main cause of vitamin D deficiency is a lack of exposure to sunlight. Additionally, patients with malabsorptive or renal disorders and patients who are taking antiretroviral drugs, anticonvulsants, and certain drugs that inhibit osteoid mineralization (aluminum, fluoride, iron, and etidronate) may also have an increased risk of developing osteomalacia.
• Normal vitamin D levels are greater than 20 ng/mL. Vitamin D deficiency is defined as serum 25(OH)D blood concentrations below 10 ng/mL and levels of 10–20 ng/mL are defined as vitamin D insufficiency. The normal value threshold is higher in the elderly population at or above 30 ng/mL.
• Vitamin D, calcium, and phosphate are all crucial in the process of bone formation. Vitamin D_3_ and vitamin D_2_ are absorbed in the bloodstream and converted through a series of steps to calcitriol, the active form of vitamin D, which regulates blood calcium levels and release of PTH which in turn influences osteoblastic and osteoclastic activity in bone. Calcitriol also has a role in calcium and phosphate absorption and utilization in various metabolic functions throughout the body.
• Severe and persistent vitamin D deficiency causes chronic hypocalcemia leading to secondary hyperparathyroidism. A sustained increase in PTH leads to an increase in bone resorption and progressive demineralization. The earliest laboratory findings are an elevated alkaline phosphate level, followed by elevated PTH levels and low calcium and vitamin D levels.
• The symptoms of rickets and osteomalacia are age-dependent. In children, where bone development along with mineralization is affected, rickets present with bone deformities such as flattened occipital bones, frontal bossing, rachitic rosary, pigeon chest, lumbar lordosis, and bowing of the legs. Adults on the other hand, where mainly mineralization of the bone is affected, present with bone pain, muscle weakness, and fragility fractures.
• The bone pathology seen in both rickets and osteomalacia is characterized by an excess of unmineralized matrix. Rachitic bones have persistence of cartilage due to poor mineralization with defective osteoid deposition. Bones in osteomalacia have defective bone remodeling and excess persistent osteoid due to reduced mineralization.
• Both disorders have weakened soft bones and a higher vulnerability to fracture due to an overdrive of osteoclast activity from the body's compensatory mechanisms to correct low blood calcium levels.
• Radiologically, X-rays of the knees and wrist show splaying, fraying, cupping, and coarse trabecular pattern of the metaphysis. Pseudofractures or Looser zones are often present in the later stages of the disorder. Radiographic findings in osteomalacia show increased osteoid thickness and volume with a mineralization lag time.
• The most important treatment for osteomalacia and rickets patients is vitamin D supplementation and dietary fortification when appropriate. Symptoms may take a few weeks to months to resolve; however, the radiological and histological changes may not improve for a few months to years.
• Osteomalacia and rickets can be prevented by increasing sun exposure to obtain adequate vitamin D levels, as well as addressing at-risk populations and supplementing before symptoms arise.

## Funding

This research received no specific grant from any funding agency in the public, commercial, or not-for-profit sectors.

## Declaration of competing interest

The authors Sarah Bunker, Judy Blebea, and Jyotsna Pandey declare no financial or other conflicts of interest.
